# Intramuscular Dirofilariasis Mimicking an Orbital Metastasis in a Patient with Breast Cancer

**DOI:** 10.1155/2012/103154

**Published:** 2012-09-13

**Authors:** Brett M. Henderson, Christopher H. Hunt, Laurence J. Eckel, Kara M. Schwartz, Felix E. Diehn, Bobbi S. Pritt, David J. Schembri Wismayer, James A. Garrity

**Affiliations:** ^1^College of Osteopathic Medicine, Des Moines University, 3200 Grand Ave. Des Moines, IA 50312, USA; ^2^Department of Radiology, Mayo Clinic, 200 1st Street SW, Rochester, MN 55905, USA; ^3^Department of Laboratory Medicine and Pathology, Mayo Clinic, 200 1st Street SW, Rochester, MN 55905, USA; ^4^Department of Ophthalmology, Mayo Clinic, 200 1st Street SW, Rochester, MN 55905, USA

## Abstract

We present the unusual case of a 74 year-old female with a history of breast cancer who presented with acute painless orbital swelling and vertical diplopia. MRI revealed a focal enhancing mass within the superior rectus muscle. As the concern for metastatic disease was high, surgical biopsy was performed and revealed an unusual mimicker of metastatic disease, the parasitic infection dirofilariasis.

## 1. Introduction

Orbital metastases can occur in approximately 8 to 10% of patients with metastatic breast cancer [1]. With the exception of carcinoid tumors, the presence of orbital metastasis is usually an ominous finding with a poor prognosis. We present the case of a patient with a history of breast cancer that presented with painless unilateral orbital swelling. The initial clinical and radiographic findings were suggestive of hemorrhage in association with a suspected varix but followup imaging was more worrisome for a metastasis involving the superior rectus muscle. At biopsy, an unexpected finding of dirofilariasis was made.

Dirofilariasis is a parasitic disease that rarely affects humans. The life cycle of this parasite normally involves dogs as a primary host and mosquitoes as potential vectors. While intraocular infections have been reported with dirofilariasis, infections involving the orbit and, in particular, the extraocular muscles are exceedingly uncommon [2–6]. 

## 2. Case Report

A 74-year-old woman was initially aware of intermittent vertical diplopia which then became constant over the next 24 hours. This was followed by painless left orbital swelling with mild erythema and swelling of the left upper lid. The globe itself was normal. Visual acuity was 20/20 right eye and 20/25 left eye. There was mild erythema and swelling of the left upper eyelid. Eyelid fissures measured 10 mm on the right and 6 mm on the left. The left globe was 1 mm proptotic. Ductions of the left eye were mildly deficient in upgaze. Initial CT and MRI imaging, along with the clinical history were suggestive of a varix. She returned 1 month later with improvement of the lid swelling and diplopia however repeat MRI was unchanged raising concern for a metastasis. At surgery, a firm yellow tan nodule was seen within the superior rectus/levator muscles. Approximately 70% of the mass was removed since it seemed to involve most of the muscle. Intraoperative frozen section evaluation revealed intense necrotizing granulomatous inflammation. 

**Figure 1:**
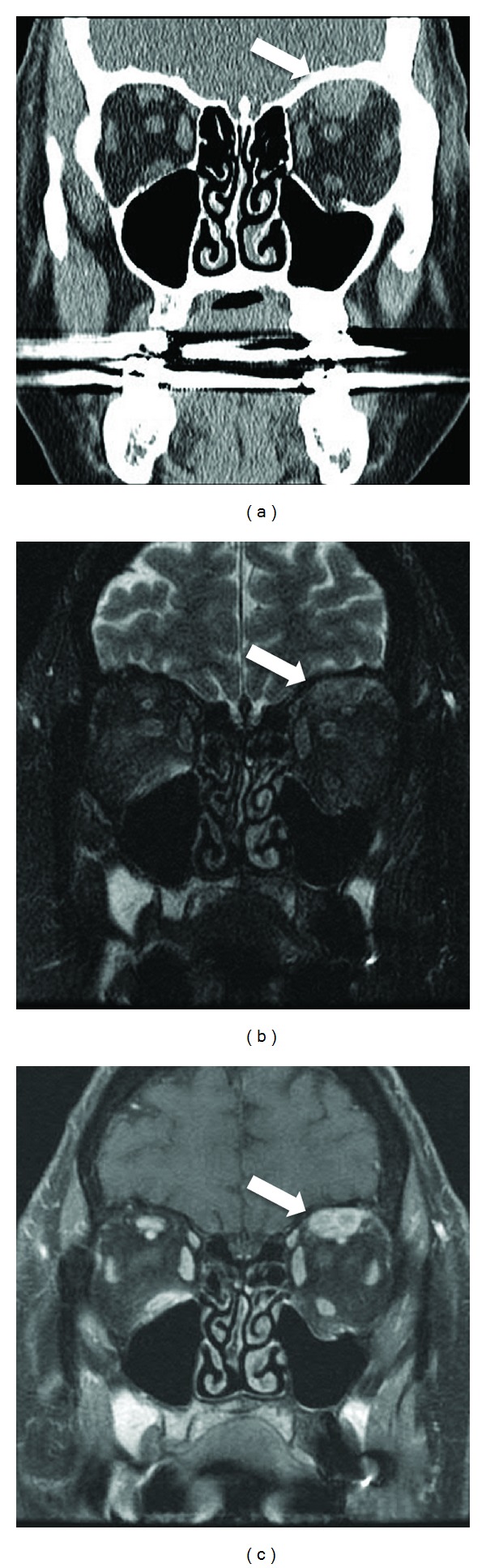
Coronal images from a noncontrast CT (a), fat-saturated T2WI (b), and fat-saturated postgadolinium T1WI (c) demonstrate a noncalcified, avidly though heterogeneously enhancing mass involving the left superior rectus muscle (arrows). Note the lack of any significant intra- or extraconal inflammatory changes in the orbital fat, and the slightly displaced but normal caliber superior ophthalmic vein along the inferior aspect of the mass.

**Figure 2:**
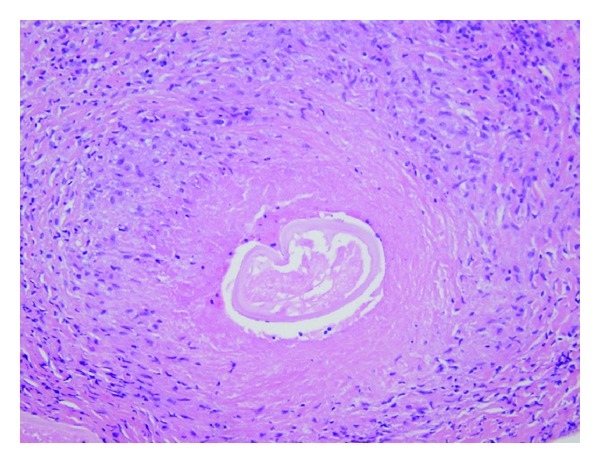
Hematoxylin and Eosin (H&E)-stained section showing a cross-section of a degenerating nematode (roundworm), consistent with *Dirofilaria* sp., surrounded by necrotizing granulomatous inflammation (200 times original magnification).

The patient owned a dog and claimed to spend significant time outdoors in her garden. MRI of the head and orbits revealed an avidly enhancing 1.3 cm maximal dimension mass in the left superior rectus muscle (Figure 1). A normal superior ophthalmic vein was identified without evidence for any inflammatory changes in the orbit fat. Of note, the patient had a history of stage I breast cancer that was treated with surgery and radiation therapy. Five-year cancer surveillance had revealed no evidence of recurrence. The differential diagnosis included metastatic disease based on MR findings versus intramuscular hematoma based on the rapid onset of symptoms. The patient was treated with antibiotics for one month for presumptive cellulitis with no change in the MRI findings. Given the persistence of the clinical symptoms and MRI findings, surgical biopsy was recommended. At surgery, a tan nodule was easily separated from the superior rectus muscle. Pathology demonstrated a necrotizing granulomatous infection with fragments of a degenerating nematode (round worm) consistent with *Dirofilaria* sp. (Figure 2). Follow-up with infectious disease revealed no evidence of more widespread systemic disease.

## 3. Discussion

Dirofilarial infections are dead-end parasitic infections in humans, normally occurring without risk of dissemination. Canines are a common primary host of *Dirofilaria* species. The microfilariae that circulate in the bloodstream of the primary host can be taken up by blood sucking insects (most commonly mosquitoes) and transmitted to humans through inoculation. The parasite has been reported to survive in human tissues for years without causing symptoms. In most human cases, the infection is discovered due to the associated immune response causing a granulomatous reaction and mass [5–7].

Dirofilaria infection in humans can occur in various tissues. Cases of orbital [2, 4, 6, 8, 9], pulmonary [10–12], and genital [7] infections have been reported. Infections have been reported to appear as a pseudotumor in the lung and scrotum on imaging studies. Reports of pulmonary dirofilariasis have described the presence of a solitary “coin” lesion on plain-film or CT images [8]; however, this finding was not seen in our case. In most cases, the infection involves painless swelling and the formation of a cyst or nodule-like lesion. Because the infection presents as a tumor-like lesion, the involved tissues are usually surgically resected [7]. In humans, reports of pulmonary dirofilariasis are more common than orbital dirofilariasis. In these cases, the parasitic infection is often mistaken for metastatic or primary cancer, usually requiring biopsy to make the diagnosis. 

Our case demonstrates how the MRI appearance of ocular dirofilariasis can mimic the appearance of metastatic disease, especially in patients with a history of systemic cancer. As the MRI imaging features are nonspecific, the tumor-like appearance of the affected tissues typically leads to surgical resection [3, 7, 10]. In the orbit, the acute, painless presentation may be a clinical feature to help suggest this infection, especially in patients with an exposure history [2, 8].
